# Repair of Aerospace Composite Structures Using Liquid Thermoplastic Resin

**DOI:** 10.3390/polym15061377

**Published:** 2023-03-10

**Authors:** Tayyab Khan, Farrukh Hafeez, Rehan Umer

**Affiliations:** 1 Department of Aerospace Engineering, Khalifa University of Science and Technology, Abu Dhabi P.O. Box 127788, United Arab Emirates; 2 Department of Mechanical Engineering, School of Engineering, University of Birmingham, Dubai International Academic City, Dubai P.O. Box 341799, United Arab Emirates

**Keywords:** scarf joints, thermoplastics, residual strength, crack healing, microstructure

## Abstract

In this study, two types of carbon-fiber-reinforced plastic (CFRP) composite scarf geometries were created using two scarf angles, i.e., 1.43° and 5.71°. The scarf joints were adhesively bonded using a novel liquid thermoplastic resin at two different temperatures. The performance of the repaired laminates was compared with pristine samples in terms of residual flexural strength using four-point bending tests. The repair quality of the laminates was examined by optical micrographs, and the failure modes after flexural tests were analyzed using a scanning electron microscope. The thermal stability of the resin was evaluated by thermogravimetric analysis (TGA), whereas the stiffness of the pristine samples was determined using dynamic mechanical analysis (DMA). The results showed that the laminates were not fully repaired under ambient conditions, and the highest recovery strength at room temperature was only 57% of the total strength exhibited by pristine laminates. Increasing the bonding temperature to an optimal repair temperature of 210 °C resulted in a significant improvement in the recovery strength. The best results were achieved for laminates with a higher scarf angle (5.71°). The highest residual flexural strength was recorded as 97% that of the pristine sample repaired at 210 °C with a scarf angle of 5.71°. The SEM micrographs showed that all the repaired samples exhibited delamination as the dominant failure mode, whereas the pristine samples exhibited dominant fiber fracture and fiber pullout failure modes. The residual strength recovered using liquid thermoplastic resin was found to be much higher than that reported for conventional epoxy adhesives.

## 1. Introduction

It has been well-established that advanced fiber-reinforced polymer composites (FRPCs) have emerged as the material of choice in multiple industries due to their superior strength, light weight, load-bearing capability, high corrosion and chemical resistance, and enhanced durability and fatigue life [1,2,3,4,5,6]. The aerospace industry is undoubtedly the primary consumer of FRPCs, and the adoption of FRPCs for both primary and secondary structures has revolutionized the modern aviation industry. The biggest aircraft manufacturers, i.e., Boeing and Airbus, are known for their gamechanger aircrafts such as the Boeing 787 and Airbus A350, comprising 50% and 53% composite materials by weight, respectively [7,8]. Similarly, FRPCs are a popular choice in the automobile industry, where adopting composite parts can not only reduce the overall weight by up to 40% but also provide around 60% reduced tooling costs along with greater flexibility in terms of design and manufacturing [9]. Among the different car manufacturers, BMW, Audi, and Toyota standout for their mass production of multiple FRPC parts [10]. Often regarded as the pinnacle of motorsport, the Formula-one (F1) industry is another noteworthy primary consumer of FRPCs, as composites have completely modernized the auto-sport industry, given that 80% of an F1 car by volume is comprised of composite materials [11]. Other notable FRPC consumers include the marine industry, the defense sector, wind turbines, and the sports industry [3].

Most of the complex structural composite parts in the aerospace and automotive industries are finalized by joining smaller components, and the joints always represent the weakest region [12]. Advancements in fusion bonding techniques have paved the way for utilizing thermoplastic composites instead of conventional thermosetting composites. Unified aerostructures such as the tail of the Augusta Westland AW169 and the wing leading edges on the Airbus A340 and Airbus A380 are only possible due to the joining capability of thermoplastic composites [8,13]. In fact, the ever-growing popularity of thermoplastic composites in the aerospace industry is primarily driven by the complexity of joining conventional thermosetting parts, the inability of thermosetting composites to be formed or melted, and their lack of recyclability [1,8,14]. Thermoplastic composites stand out due to their impressive properties compared to their thermosetting counterparts, such as the high impact toughness, faster processability, higher service temperatures, less environmental impact, recyclability, and unlimited shelf life of their source materials [15,16]. Additionally, it is critically important to develop systematic repair methodologies for composite parts when they are damaged. In most applications, especially in the aerospace industry, composite structures are often removed or replaced. The cost of repairing thermoset FRPC aerostructures ranges from USD 15,000 to 150,000 per part, which only constitutes around 8–25% of the cost it would take to replace them [8]. Therefore, viable repair techniques can not only make the product life cycle more sustainable but are also less expensive compared to part replacement.

Multiple composite repair methods have been adopted in the past, such as mechanical fastening, adhesive bonded repairs, fusion bonding, and crack healing (autohesion) [1]. Among these composite repair techniques, scarf repair is widely preferred, as it results in less severe stress concentration, can restore the load-carrying capacity of the damaged part to its as-designed strength, and minimizes the disruption of aerodynamic surfaces in aerostructures [17,18]. Scarf repairs have achieved the highest levels of strength recoveries, between 70 and 100%, with minimal surface changes [19]. Scarf repair angles vary between 3° and 7°; however, a number of studies have reported in the literature that the optimum repair angle is around 3 to 5°, i.e., a 20:1 taper angle [20,21,22,23]. Conventionally, an epoxy adhesive is used for repairing scarf joints. As is well-established, the adhesive bonding of composite laminates, especially in the form of scarf joints, offers incomparable advantages, such as uniform stress distribution throughout the joint, superior mechanical performance and strength, minimalistic changes to the parent structure, a low cost, and suitability for the bonding of complex composite structures [24,25]. Owing to these advantages, adhesive bonding has become the primary joining technique for CFRP composites in the aerospace industry [26]. The strength of the repaired composite laminates is generally evaluated in terms of the residual strength of the pristine laminates without repair. It is worth mentioning that the four-point bending approach is often regarded as the best method for screening repair techniques and investigating the residual strength of the repaired laminates, as it is not affected by loading anomalies [27].

In recent years, considerable efforts have been made to shift towards sustainability in the composites industry, and one of the primary research areas in this context has been the development of liquid thermoplastic resins and adhesives that can be cured at room temperature, such as Elium^®^ resin [28]. In contrast to other thermoplastic resins, Elium^®^ is a liquid resin with a viscosity comparable to that of conventional thermoplastic resins (i.e., 0.1 Pa.s.), which makes it not only compatible with liquid composite molding techniques but also able to be cured at room temperature [1]. Numerous studies have already been conducted on the cure kinetics, mechanical performance, recyclability, and ultrasonic welding of innovative thermoplastic resins, as summarized in a comprehensive review article [29]. Recently, Khan et al. [1,30] explored the joining capability of liquid thermoplastic resin and demonstrated that the flexural strength of the artificially damaged laminates could be recovered to as high as 96% of the pristine specimens. Similarly, the interlaminar fracture toughness of the CFRP composite laminates healed using liquid thermoplastic resin was reported to be 1.16 N/m, much higher than that recorded for conventional epoxy adhesives [1].

In this study, the feasibility of repairing CFRP composite-based scarf joints using liquid thermoplastic resin was explored by creating two different scarf geometries with scarf angles of 1.43° and 5.71° and repairing them at both room temperature and at an optimum repair temperature for Elium^®^ resin, i.e., at 210 °C. The quality of the scarf surfaces after machining and that of the repaired specimens were evaluated through optical micrographs. The thermal stability of the Elium^®^ resin was studied through thermogravimetric analysis (TGA), whereas the stiffness of the composite laminates was assessed through dynamic mechanical analysis (DMA). The quality of the repaired samples was evaluated through four-point bending tests and by assessing their residual strength compared to the pristine samples. The failure modes in all the repaired and pristine specimens were examined through a comprehensive scanning electron microscopy (SEM) analysis.

## 2. Materials and Methods

### 2.1. Materials

The composite specimens were manufactured using the aerospace-grade “CYCOM^®^ 937A” plain-weave, woven carbon-fiber prepreg with epoxy resin as the matrix, manufactured by Solvay industries Inc. The properties of the prepreg material are provided in Table 1. The prepreg was premixed with toughened epoxy resin, and curing in either an autoclave or through hot-press molding was recommended. The epoxy resin had a service temperature of 177 °C. The carbon fibers had an areal density of 230 g/m^2^, whereas the average diameter of a single carbon fiber was 7 μm. The scarf laminates were repaired using a low-viscosity infusible thermoplastic resin, i.e., Elium^®^ 188 O, supplied by Arkema, Shanghai, China. Luperox^®^. ATC50 benzoyl peroxide (BPO) supplied by Sigma-Aldrich Corporation, St. Louis, MO, USA, was used as the initiator for the polymerization of the thermoplastic resin. Elium^®^ is a polymethyl methacrylate (PMMA)-based liquid resin that undergoes radical polymerization at an ambient temperature, initiated by the reaction of a peroxide hardener with the monomer, i.e., methyl methacrylate (MMA).

### 2.2. Methods

#### 2.2.1. Manufacturing of Composite Laminates

The laminates were manufactured by the hot compression molding process using a 120 Ton pneumatic press (model: CoexpairTM P005-1.0 × 0.65), Namur, Belgium, with a platen size of 1000 mm × 650 mm. In total, 16 layers of Cycom 937A prepreg were laid in 0°/90° orientations on a flat steel mold of dimensions 600 mm × 400 mm with spacers to control the thickness at 5.2 mm. A pressure of 8 bar was applied to consolidate the composites. The size of the manufactured laminates was 600 mm × 400 mm. The prepregs were cured following the manufacturer’s recommended cure cycle, i.e., 177 °C for two hours.

#### 2.2.2. Machining of Composite Laminates

**Figure 1:**
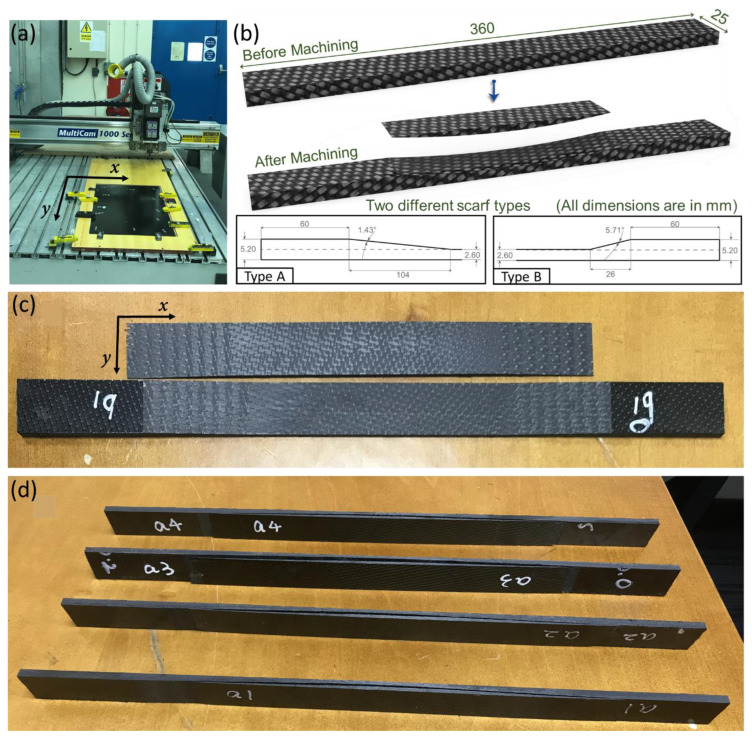
(**a**) CNC router used for the machining of the scarf joints and (**b**) the dimensions of two different scarf geometries. (**c**) A sample specimen with machined surfaces, (**d**) a set of machined samples ready for joining.

The scarf geometries were created by conducting the edge trimming technique using a 3-axis CNC router (i.e., MultiCam 1000 series), Texas, TX, USA. (Figure 1a). Initially, a 600 × 400 mm^2^ CFRP laminate with a thickness of 5.2 mm was clamped on the router. A customized wooden fixture was created for clamping the CFRP laminate by making a recess in a wooden block. The recess in the fixture was of the same size as the laminate for safe operation and quality specimens. The laminate was dropped in this recess such that the top surface was almost parallel to the border of the fixture. Clamps were installed with self-tapping bolts on the border around the recess and were tightened to hold the laminate. The flatness of the fixture was ensured while machining the recess for the profile consistency. The laminate was aligned along the axis of the router bed such that the weft was along the router gantry (the *x*-axis of the router) and the warp was aligned along the depth axis perpendicular to the gantry (the *y*-axis of the router). The profiles for the scarf surfaces were then machined for the total width required for all specimens, with margins for cutting the final width of each specimen. Four different profiles were machined, two for each type of scarf geometry. Specimens were produced by cutting them out from the laminate along the weft direction. The depth of the final cut was slightly greater than the thickness of the laminate to ensure the consistency and quality of the specimens, particularly around the edges. This resulted in grooves in the wooden fixture; however, the fixture eliminated any possibility of damage to the cutting tool or the machine bed.

The edge of the laminate was trimmed in a climb milling configuration using a 10 mm diamond solid-carbide-coated segmented straight flute tool. The scarf surfaces were created by trimming at a cutting speed of 4000 rpm and a feed rate of 800/min, as recommended by the cutting tool manufacturer. Two different types of scarf geometry were cut using two different scarf angles, i.e., 1.43° (Type A) and 5.71° (Type B) (Figure 1b). The maximum depth of the scarf geometries was kept constant in both types of specimens at 2.6 mm. The dimensions and scarf geometries of both types of specimens are shown in Figure 1b. The experimental setup and the details of the cutting tools as well as the machining of the CFRP panels can be found elsewhere [32].

##### Surface Roughness

**Figure 2:**
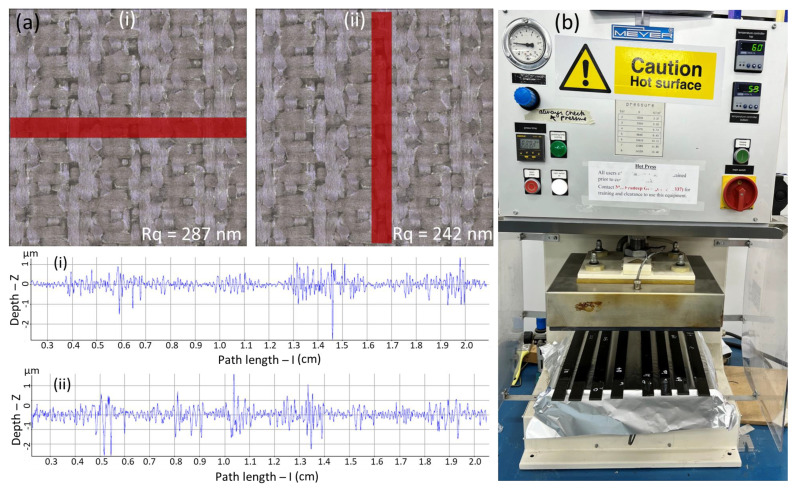
(**a**) Surface roughness profile of the specimens and (**b**) hydraulic press used for the repair of the scarf joints. Note: (i) shows roughness in the *x* direction and (ii) shows roughness in the *y* direction.

The surface roughness of the scarf surfaces was investigated using an Alicona InfiniteFocus optical 3D measuring instrument, Alicona, Graz, Austria, with a vertical resolution as high as 10 nm. A comprehensive roughness analysis was conducted by observing the roughness profile in both the *x* and *y* directions. The root mean square (RMS) value is commonly used to calculate the average surface roughness using data variance [33]. The Rq (RMS) in the *x* direction was found to be 287 nm and was recorded as 242 nm in the *y* direction, as shown in Figure 2a. Both types of specimens exhibited a similar roughness profile.

#### 2.2.3. Repair of Scarf Joints

The composite laminates were repaired using a liquid thermoplastic resin. The specimens were repaired at both ambient temperature as well as at 210 °C using a Meyer^®^ hot platen press (model: APV 3530), Roetz, Germany, equipped with dual-contact heating platens presenting a total platen area of 350 × 300 mm^2^ (Figure 2b). It is important to note that the specimens repaired at both ambient temperature and at 210 °C were repaired at a constant pressure of 4 bar. The specimens repaired at room temperature were left to cure for 24 h, whereas the specimens repaired at 210 °C were left to cure for 90 min. The repair temperature and the repair time were selected based on the design of the experiments and the analysis of variance, and the optimum repair temperature and time for liquid thermoplastic resin were found to be 210 °C and 90 min, respectively [1,30]. The thermoplastic resin was mixed with 3 wt.% BPO and stirred for 5 min to initiate the polymerization. The resin was then carefully applied on both surfaces of the scarf geometries, and the specimens were placed between the platens of the Meyer^®^ press. An aluminum foil was placed on both sides of the specimens to avoid direct contact with the heating plates. Additionally, a thermocouple was used to monitor the actual repair temperature. The quality of the repaired laminates was investigated through microscopic visualizations and by subjecting them to flexural tests. The repair performance was evaluated in terms of the residual strength compared to that of the pristine specimens without any joints.

#### 2.2.4. Optical Microscopy

The quality of the scarf surfaces and the repaired specimens was investigated through optical images using a Leica DMS100^®^ digital microscope, Wetzlar, Germany, capable of taking images at 300× magnification. The surface and the cross-sectional images of the scarf geometries were thoroughly observed for any machining damage. Once repaired, the quality of the scarf joints was analyzed based on cross-sectional images throughout the scarf length of the samples.

#### 2.2.5. Thermogravimetric Analysis

The TGA of liquid thermoplastic resin was performed using the TA SDT Q600 instrument, New Castle, DE, USA, to analyze the thermal stability of the thermoplastic resin and identify the repair and critical degradation temperatures. Multiple TGA scans were performed under a nitrogen environment at a purge flow rate of 20 mL/min and a heating rate of 10 °C/min. The analysis was performed from 30 °C to 1000 °C using alumina pans, and the mass for the specimens was approximately 10 mg.

#### 2.2.6. Dynamic Mechanical Analysis (DMA)

The DMA scans were performed using a NETZSCH DMA 242E Artemis machine, Bayern, Germany. The DMA scans were performed in 3-point bending clamps using the temperature ramp sequence, and the span was kept constant at 40 mm. Multiple scans were performed under standard conditions by varying the temperature from 30 °C to 300 °C, and the heating rate was kept constant at 3 °C/min. The applied frequency and the dynamic amplitude were also kept constant at 1 Hz and 15 µm, respectively. The tests were conducted in accordance with the ASTM D5023-15 test standard [34].

#### 2.2.7. Flexural Testing

**Figure 3:**
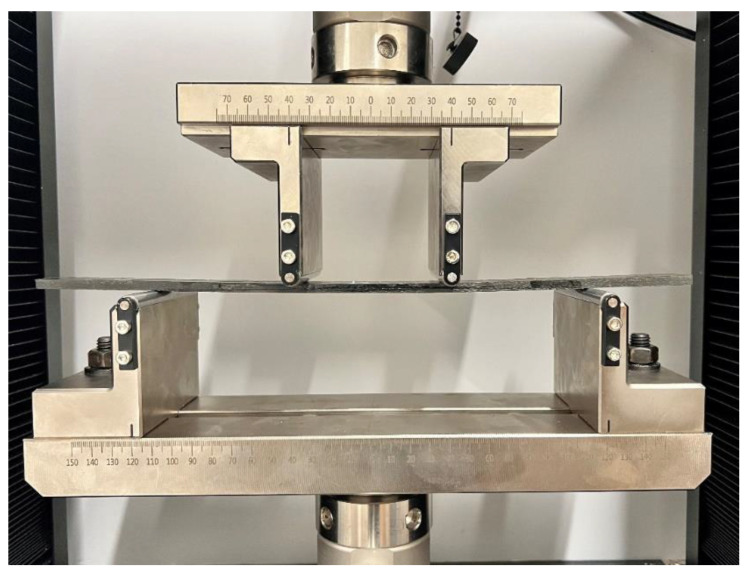
Repaired specimen under four-point bending test.

The flexural properties of the repaired composite samples were investigated using 4-point bending tests. The tests were performed on a SATL ST-1004 Universal Testing Machine (UTM), Incheon, Republic of Korea, equipped with a load cell of 2 kN. The tests were performed in accordance with the ASTM D6272 test standard [35]. The loading span and the support span were kept constant at 80 mm and 240 mm, respectively. The loading rate was kept constant at 2 mm/min, and the span-to-thickness ratio was 40:1. At least four specimens were tested for each configuration, and the variation in the results was determined through the 95% confidence interval (CI) formula, i.e., ±1.96 × *SD*)/√*n*, where *SD* is the standard deviation and *n* is the number of specimens [36]. The performance of the repaired samples was determined in terms of residual flexural strength compared to the pristine composites without any repairs. The scarf joints were placed on the bottom side (i.e., tension area of the sample) during all the tests to observe complete delamination. A repaired specimen under the 4-point bending test is shown in Figure 3.

#### 2.2.8. Fractographic Analysis

The microstructure of the repaired and pristine samples after flexural testing was analyzed using SEM analysis to correlate the flexural performance and repair quality with the failure modes. The SEM images were obtained using an FEI Quanta 250 FEG-SEM microscope, Hillsboro, OR, USA. The SEM micrographs were also correlated with recent findings in the literature on different failure modes in CFRP composite laminates.

## 3. Results and Discussion

### 3.1. Optical Microscopy

**Figure 4:**
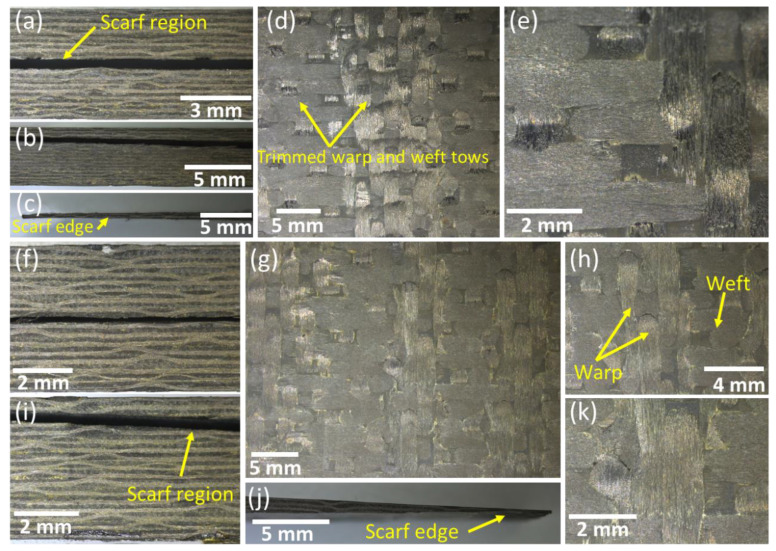
Optical micrographs of the scarf geometries (**a**–**e**) Type A and (**f**–**k**) Type B.

The surfaces of the scarf geometries and their cross-sections were examined using optical microscopy after machining. Similarly, the repair quality of the samples at both ambient temperature and 210 °C was also investigated through optical microscopy. The optical micrographs of the Type A composite laminate after machining are shown in Figure 4a–e, whereas those of the Type B samples are shown in Figure 4f–k. The micrographs showed no signs of micro/macro defects as a result of the machining process in both types of specimens. The edges of the scarf regions for both Type A (Figure 4c) and Type B (Figure 4j) were also machined precisely and had no visible damage. Apart from this, the micrographs of the laminates before the repair process also showed that there were no visible process-induced defects associated with the manufacturing of composites.

**Figure 5:**
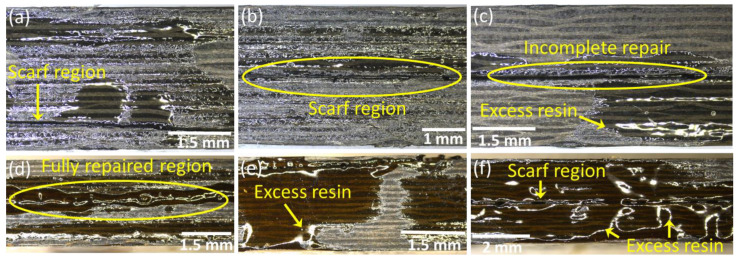
Optical micrographs of the specimens (**a**–**c**) repaired at room temperature and (**d**–**f**) repaired at 210 °C.

Both Type A and Type B specimens with different scarf geometries were initially repaired at room temperature using liquid thermoplastic resin. The thermoplastic resin was evenly applied on both surfaces of the scarf geometries, and the laminates were carefully joined. The specimens were then placed in the Meyer^®^ press under a constant pressure of 4 bar and left to cure at room temperature for 24 h. The optical images of the Type A specimens repaired at room temperature using liquid thermoplastic resin are shown in Figure 5a–c. It can be observed that the samples were not fully repaired, and the scarf regions remained open in some areas. The excess resin applied on the surfaces of the laminates was squeezed out through the cracks. Even though a constant pressure of 4 bar was applied on the specimens, the laminates exhibited a random repair pattern comprising fully repaired as well as incomplete repair regions. These incomplete repairs had a major influence on the mechanical performance of the repaired laminates, as discussed in detail in Section 3.4.

Once the repair temperature was increased to 210 °C, the cross-sectional micrographs showed better bonding by the liquid thermoplastic resin, and the laminates were observed to be fully repaired (Figure 5d–f). Again, the excess resin was squeezed out of the cracks due to the applied pressure. It is important to note that even at a high temperature such as 210 °C, there was no visible deformation or flow in the thermosetting resin used for manufacturing the composite laminates in the form of prepregs. Therefore, no physical or chemical changes were made to the parent structure during the repair of the scarf joints using liquid thermoplastic resin. Based on the high quality of the repairs, both types of composite laminate repaired at 210 °C exhibited significant improvements in their mechanical performance, as discussed in detail in Section 3.4.

### 3.2. Thermogravimetric Analysis

**Figure 6:**
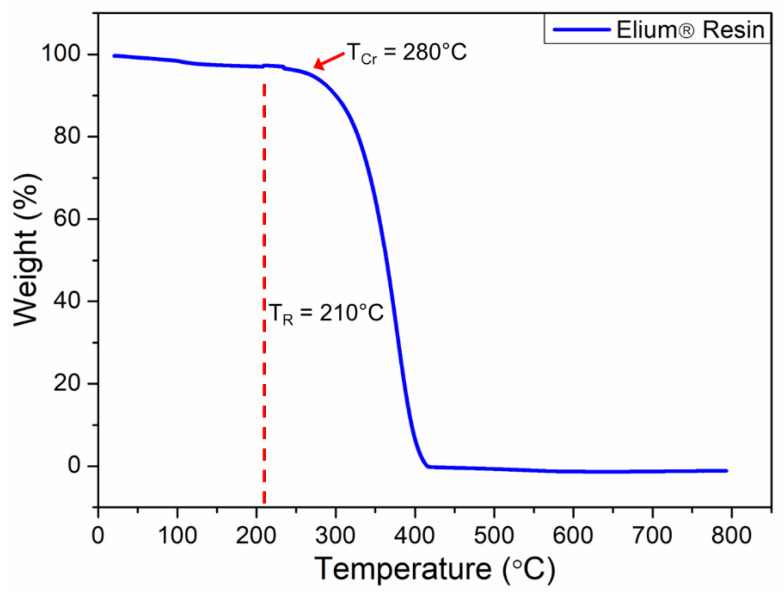
Thermal degradation of the liquid thermoplastic resin.

A TGA analysis of the liquid thermoplastic resin was completed to identify the critical degradation temperature (T_cr_) of the liquid thermoplastic resin. Multiple TGA scans of the pure liquid thermoplastic resin were performed by keeping the overall BPO content constant at 3 wt.%. As shown in Figure 6, the liquid thermoplastic resin presented a rapid degradation beyond 280 °C. Therefore, the repair temperature (T_R_) for the composite laminates was kept below T_cr_. Furthermore, the optimum repair temperature was identified as 210 °C based on a comprehensive analysis of variance (ANOVA) from a previous study on the self-healing of liquid thermoplastic composite laminates [1]. Another study has also shown that the best mechanical performance of self-healed liquid thermoplastic composites was achieved at 210 °C [30]. It is important to note that the initial drop in the weight of the resin from 100 to around 95% before reaching 280 °C was due to the dehydration process. The actual degradation of the resin started beyond this point and was reflected by the sharp drop in weight. The degradation of the resin was completed at around 420 °C, where the weight dropped to zero.

### 3.3. Dynamic Mechanical Analysis

**Figure 7:**
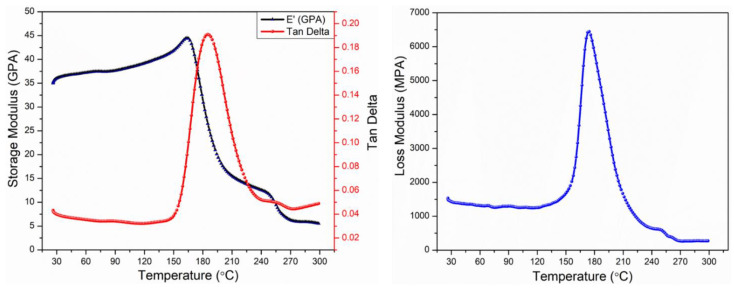
Thermomechanical properties of the pristine specimens: (**a**) storage modulus and tan delta, (**b**) loss modulus.

The thermomechanical properties of pristine composite laminates were investigated by conducting a number of DMA tests. Within the scope of these tests, the storage modulus, loss modulus, tan delta, and glass transition temperature of the composites were calculated. The average thermomechanical properties of the pristine samples are listed in Table 2. The storage modulus and tan delta over the temperature range of 30–300 °C are graphically presented in Figure 7a, whereas the loss modulus is shown in Figure 7b. The storage modulus of the specimens gradually increased as the temperature was increased before the onset of the glass transition temperature (T_g_). This initial increase in the stiffness was attributed to the thickness of the specimens. A further increase in the temperature above T_g_ led to a sudden decrease in the storage modulus due to the transition from glass to the rubbery state. The highest value of the storage modulus was recorded as 44.5 GPa in the glassy state, where the polymer chains were densely packed and experienced strong intermolecular forces. The T_g_ was calculated from the peak of the tan delta as 186.6 °C, which was comparable with the T_g_ of the epoxy resin provided in the datasheet (i.e., 190 °C).

### 3.4. Flexural Properties

**Figure 8:**
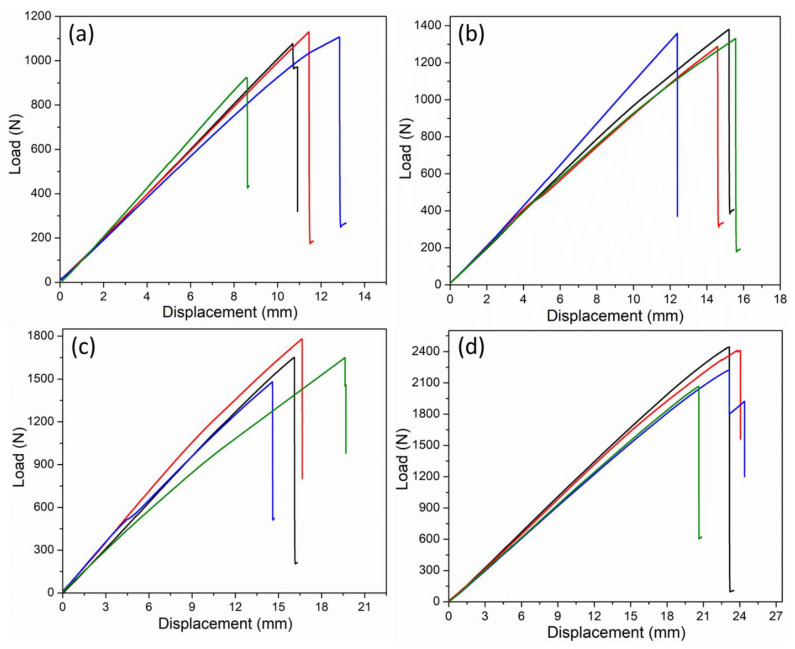
Flexural load vs. displacement curves for (**a**) Type A specimens repaired at room temperature, (**b**) Type B specimens repaired at room temperature, (**c**) Type A specimens repaired at 210 °C, and (**d**) Type B specimens repaired at 210 °C.

The repair performance of both types of scarf geometry was thoroughly evaluated using four-point bending tests. It was shown in a previous study that four-point bending tests can be effectively used to evaluate the residual strength of composite laminates repaired using liquid thermoplastic resin [30]. Initially, the specimens cured at ambient temperature were subjected to flexural tests. The typical load vs. displacement curves for both Type A and Type B specimens repaired at ambient temperature are shown in Figure 8a,b. The results showed that the Type A specimens with a lower scarf angle exhibited lower peak loads compared to the Type B specimens. The flexural strength for the Type A specimens repaired at room temperature was recorded as 407 MPa, whereas that of the Type B specimens was found to be 514 MPa. Therefore, an increase in the scarf angle of the repaired specimens from 1.43° to 5.71° resulted in an approximately 26% increase in the flexural strength. Both types of curves reveal a linear increase in load followed by a catastrophic failure due to the complete delamination of the scarf joint.

**Figure 9:**
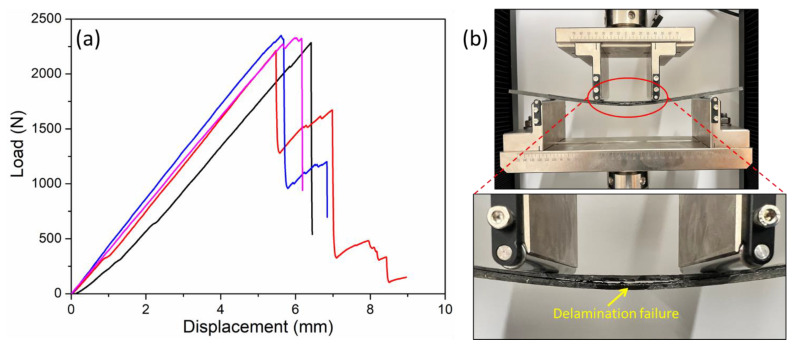
(**a**) Load vs. displacement curves for the pristine specimens and (**b**) repaired composite laminates under four-point bending tests.

Figure 8c,d shows that repairing at 210 °C had a significant impact on the overall performance of the composite laminates. The peak load carried by the Type A specimens at 210 °C increased from 1059 N to 1640 N. Similarly, the peak load carried by the Type B specimens repaired at 210 °C exhibited a dramatic increase from 1339 N to 2285 N. In terms of the flexural strength, the Type A specimens repaired at 210 °C exhibited an increase of around 55% compared to that of the same specimens repaired at room temperature. Similarly, the flexural strength of the Type B specimens increased from 514 MPa to 877 MPa (an increase of over 70%) after repairing them at 210 °C instead of ambient temperature. It is also important to note that the Type B specimens with a larger scarf angle again exhibited higher flexural strength compared to the Type A specimens with a smaller scarf angle. The Type A specimens repaired at 210 °C exhibited around 40% higher flexural strength (877 MPa) compared to that of the Type A specimens (630 MPa) repaired at the same temperature. It has been well-established in the literature that increasing the repair temperature of the liquid thermoplastic resin leads to a significant improvement in the mechanical performance of the repaired laminates [1,30]. The cross-sectional images also revealed that specimens were fully repaired at 210 °C (Figure 5). As mentioned before, the optimum repair temperature was selected based on a comprehensive experimental design technique as well as a detailed analysis of variance conducted in a previous study [1]. A typical specimen upon failure under a four-point bending test is shown in Figure 9b. Based on Figure 9b, failure in all the specimens initiated from the central scarf region and rapidly propagated through the edges, causing complete delamination.

The repair quality of all the composite specimens repaired at both room temperature and at 210 °C was evaluated by comparing the results with the pristine specimens without any scarf joints. The pristine specimens were also subjected to flexural tests, and the corresponding load vs. displacement curves are shown in Figure 9a. The flexural properties of the pristine specimens and the repaired laminates in terms of peak load, flexural strength, residual strength, and flexural modulus are presented in Table 3. As expected, the best performance in terms of flexural properties was exhibited by the pristine specimens. The highest average value of the flexural strength presented by the pristine specimens was recorded as 901 MPa. Furthermore, Figure 9a shows that the pristine specimens also exhibited different load–displacement curves compared to the repaired specimens. This difference was primarily associated with the different failure modes observed in the pristine specimens, as discussed in detail in Section 3.5.

A comparison of the flexural strengths of the repaired samples with that of the pristine samples showed that the lowest value of the residual strength was observed as 45% for the Type A specimens repaired at ambient temperature. Similarly, the residual strength of the Type B specimens repaired at room temperature was only 57% of the total flexural strength exhibited by the pristine specimens. The residual flexural strength was significantly increased for both types of specimens by repairing them at 210 °C. In this context, the residual strength of the Type A specimens repaired at 210 °C was 70% of the flexural strength shown by the pristine samples. The highest average residual strength, which was exhibited by the Type B specimens repaired at 210 °C, was found to be 97% that of the pristine specimens. It can be noted that the Type B specimens repaired at 210 °C exhibited similar flexural strength to that recorded for the pristine specimens, indicating the high quality of the scarf repair technique using the liquid thermoplastic resin.

A comparison of the results with the literature revealed that liquid thermoplastic resin could be effectively utilized for the adhesive repair of scarf joints to achieve better mechanical properties compared to conventional structural adhesives. For example, Khashaba et al. [37] conducted a similar study to investigate the flexural performance of scarf-repaired CFRP composite laminates using epoxy adhesives. The flexural performance of the adhesively bonded scarf joints was investigated through three-point bending tests. The highest residual flexural strength was recorded as 86.1%. In order to further recover the residual strength, the epoxy adhesive was modified with multi-walled carbon nanotubes (MWCNTs). The residual strength of the specimens repaired using the MWCNT-modified adhesive was further increased to 98.2%. Nevertheless, only a single scarf angle of 15° was considered in the study. Other studies in the literature have shown that the performance of composite laminates repaired using adhesives can also be improved by modifying the adhesive with nanoparticles such as MWCNTs, SiC, and Al_2_O_3_ [38]. Atas et al. [39] used the hand layup technique as well as the resin infusion process to repair scarf joints in CFRP composites. The results showed that the hand layup technique could only recover around 30% of the flexural strength of the pristine specimens. The residual strength of the specimens repaired using the infusion process was improved compared to that of the specimens repaired using the hand layup technique. However, the highest strength recovery was limited to only around 50% of the strength exhibited by the pristine specimens.

In terms of the effect of the scarf angle on the residual strength, Khashaba et al. [40] conducted a comprehensive study using different scarf angles, including 5, 10, 15, 30, and 45°, and investigated their effect on the tensile residual strength. Both the experimental and numerical tests showed that the highest tensile strength was achieved for the repaired specimens with a 5° scarf angle. In another study [41], it was demonstrated that the best performance in terms of tensile strength as well as fatigue strength was achieved for a 5° scarf angle.

A thorough literature review revealed that the results obtained in this study were in line with the recent findings on the subject, and liquid thermoplastic resin could be effectively utilized to restore the as-manufactured mechanical properties without any modification or surface changes in the parent structure. Furthermore, the proposed methodology could be effectively utilized to repair different kinds of damaged aerostructures, such as impact-damaged laminates, which could be investigated in a future study. Patches of Elium^®^ composites could be directly applied to the damaged structures in the form of other joints such as lap joints and stepped joints. In other scenarios, Elium^®^ resin could also be directly impregnated and cured at relatively low temperatures or even at room temperature to repair matrix damage. It is important to note that Elium^®^ is also compatible with thermoplastic welding techniques such as ultrasonic welding. In this context, there is a lot of potential for adopting welding repair techniques using Elium^®^ composites. Most importantly, the proposed technique could offer solutions for a number of existing challenges in both the aerospace and automotive industries, such as the requirements for high consolidation pressures for conventional repairing techniques, tedious surface preparations, specialized equipment, and high repair temperatures.

### 3.5. Fractographic Analysis

**Figure 10:**
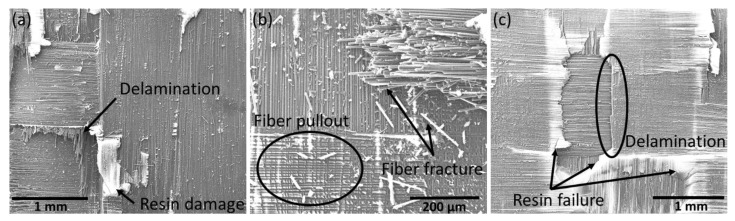
SEM micrographs showing different failure modes in the samples repaired at room temperature after flexural testing (**a**) delamination, (**b**) fiber pullout and fracture and (**c**) resin failure and delamination.

The results in Section 3.4 demonstrated that even though the strength of the repaired samples was recovered to up to 97% that of the pristine samples, all the repaired specimens exhibited a similar delamination failure mode. The failure of the scarf-repaired specimens at both room temperature and 210 °C after flexural testing was thoroughly investigated through SEM micrographs. Both Type A and Type B specimens exhibited similar failure modes; therefore, only the fractographic analysis for the Type A specimens is considered here. The SEM images of the Type A specimens repaired at ambient temperature are shown in Figure 10. As expected, the failure at the microscopic level was mainly comprised of delamination at the tow level. Apart from the delamination failure mode, rigorous fiber fracture was also observed throughout the delaminated surfaces (Figure 10b). Upon failure, multiple fibers were also pulled out of the specimens. Along with this, excessive liquid thermoplastic resin failure was observed across the edges of individual fiber tows, promoting the delamination failure. The SEM micrographs also revealed a fiber–matrix interface failure mode, especially in the fiber fracture regions. Nonetheless, macroscopic delamination was the most dominant failure mode in both Type A and Type B specimens repaired at room temperature, due to the incomplete scarf repair.

**Figure 11:**
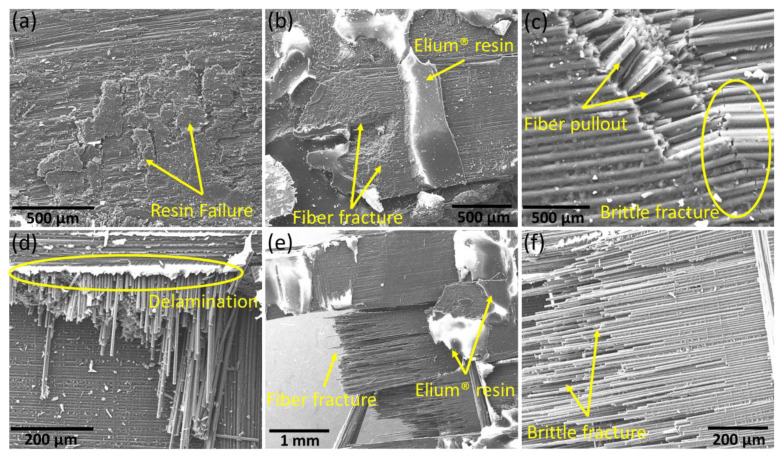
SEM micrographs showing different failure modes in the samples repaired at 210 °C after flexural testing (**a**–**c**) Type A and (**d**–**f**) Type B specimens.

As mentioned before, increasing the repair temperature to 210 °C significantly influenced the flexural properties of the repaired specimens and hence their microstructures upon failure. The SEM micrographs of the Type A and Type B specimens repaired at 210 °C upon failure after flexural testing are shown in Figure 11a–c and Figure 11d–f, respectively. The failure comprised a dominant delamination mode along with resin damage and fiber fracture. The micrographs showed a stronger bond between the liquid thermoplastic resin and the composite laminates. The failure was mainly comprised of bulk liquid thermoplastic resin failure in the form of patches on both the top and bottom sides of the repaired laminates. Along with this, the fibers were also pulled out and fractured upon the complete failure of the scarf joints, as the scarf geometry was pulled out due to complete delamination. The carbon fibers exhibited the commonly observed brittle fracture mode, especially along the edges of the scarf joints [42].

**Figure 12:**
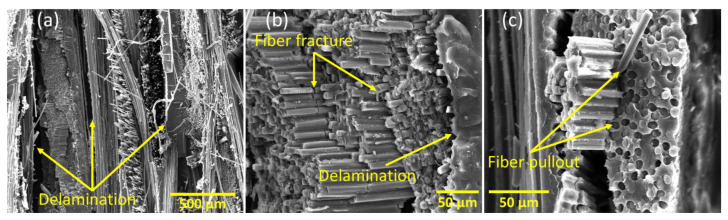
SEM micrographs showing different failure modes in the pristine specimens (**a**) delamination, (**b**) fiber fracture and (**c**) fiber pullout.

The failure of the pristine specimens without any scarf joints was also investigated at a microscopic level after subjecting them to flexural tests. The SEM micrographs in Figure 12 showed that the pristine CFRP specimens exhibited a dominant brittle fiber fracture failure mode commonly observed in carbon-fiber-based composite laminates [3]. Apart from that, the microscopic failure comprised delamination, fiber pull out, fiber cracking, and resin damage. Numerous studies in the literature have observed similar failure modes in CFRP composite specimens [42,43,44,45]. The results also showed a strong interface between the fibers and the thermosetting matrix, which was primarily due to the high surface roughness of the carbon fibers. As a result of this surface roughness, the surface area per unit volume of the fibers increased, and a stronger bond was established due to mechanical interlocking [3]. Based on this strong bonding, most of the fractured regions showed the collective brittle failure of carbon fibers and the matrix.

## 4. Conclusions

In this article, CFRP composite laminates were manufactured, and two different types of scarf geometry were created through precise machining. Two different scarf angles, i.e., 1.43° (Type A) and 5.71° (Type B), were created, and the specimens were repaired using a novel liquid thermoplastic resin at both room temperature and at 210 °C. The quality of the scarf surfaces as well as the repair quality of the laminates was examined through optical microscopy. The mechanical performance of the repaired laminates was evaluated in terms of the residual flexural strength compared to that of the pristine specimens by subjecting them to four-point bending tests. The thermal stability of the thermoplastic resin was investigated through TGA analysis, whereas the stiffness of the pristine specimens was calculated through DMA. Furthermore, the failure modes in all the repaired specimens as well as in the pristine specimens were examined through SEM analysis.

The optical micrographs showed incomplete repair in both types of joints at room temperature, whereas the laminates were observed to be fully repaired at 210 °C. As a result, the specimens repaired at 210 °C exhibited superior mechanical properties compared to those repaired at room temperature. The highest residual flexural strength was recorded as 97% that of the pristine specimens for the Type B specimens repaired at 210 °C. The lowest residual strength (47%) was recovered for the Type A specimens repaired at room temperature. For repair at both room temperature and 210 °C, the best performance was exhibited by the Type B specimens. Therefore, the optimum results were achieved for a scarf angle of 5.71°, as also reported by other researchers. A comparison with the recent findings on the subject revealed that scarf repair using a liquid thermoplastic recovered much higher residual flexural strength than other epoxy adhesives. The SEM micrographs showed that all the repaired laminates demonstrated a dominant buckling failure mode, whereas the pristine specimens showed dominant fiber fracture and fiber pull out failure modes.

## Figures and Tables

**Table 1:** Properties of the prepreg used in this study [31].

Property	Description
Name	CYCOM^®^ 937A
Fiber type	Carbon fiber
Weave type	Plain
Fiber count	3 K
Fiber diameter (µm)	7
Density (g/cm^3^)	1.76
Areal weight (g/m^2^)	230
Resin type	Toughened epoxy
Resin density (g/cm^3^)	1.27
Glass transition (°C)	190
Nominal thickness (mm)	0.36

**Table 2:** Viscoelastic properties of pristine specimens made from epoxy-resin-based prepregs.

Sample	Viscoelastic Properties			
	Maximum Storage Modulus (GPa)	Maximum Loss Modulus (GPa)	Tan Delta Peak	T_g_ (°C)
Pristine	44.5	6.5	0.19	186.6

**Table 3:** Average flexural properties of the pristine and repaired specimens.

Results	Pristine	Specimen Type			
		Repaired (25 °C)		Repaired (210 °C)	
		Type A	Type B	Type A	Type B
Peak Load (N)	2356 ^± 96^	1059 ^± 22^	1339 ^± 34^	1640 ^± 106^	2285 ^± 152^
Flexural strength (MPa)	901 ^± 21^	407 ^± 31^	514 ^± 13^	630 ^± 41^	877 ^± 58^
Residual strength (%)	--	45	57	70	97
Flexural modulus (GPa)	65 ^± 1.5^	30 ^± 1.9^	36 ^± 1.1^	47 ^± 2.3^	64 ^± 2.5^

## Data Availability

All the data used to support the findings of this study are included within the article.

## References

[1] Khan T., Irfan M.S., Cantwell W.J., Umer R. (2022). Crack healing in infusible thermoplastic composite laminates. Compos. Part A Appl. Sci. Manuf..

[2] Parmar H., Khan T., Tucci F., Umer R., Carlone P. (2022). Advanced robotics and additive manufacturing of composites: Towards a new era in Industry 4.0. Mater. Manuf. Process..

[3] Khan T., Fikri A., Irfan M.S., Gunister E., Umer R. (2020). The effect of hybridization on microstructure and thermo-mechanical properties of composites reinforced with different weaves of glass and carbon fabrics. J. Compos. Mater..

[4] Irfan M.S., Alia R.A., Khan T., Cantwell W.J., Umer R. (2023). Time-temperature superposition of flexural creep response of carbon fiber PEKK composites manufactured using different prepreg stacking sequence. J. Thermoplast. Compos. Mater..

[5] Irfan M.S., Khan T., Hussain T., Liao K., Umer R. (2021). Carbon coated piezoresistive fiber sensors: From process monitoring to structural health monitoring of composites—A review. Compos. Part A Appl. Sci. Manuf..

[6] Umer R., Waggy E.M., Haq M., Loos A.C. (2012). Experimental and numerical characterizations of flexural behavior of VARTM-infused composite sandwich structures. J. Reinf. Plast. Compos..

[7] Marsh G. (2010). Airbus A350 XWB Update. Reinf. Plast..

[8] Barroeta Robles J., Dubé M., Hubert P., Yousefpour A. (2022). Repair of thermoplastic composites: An overview. Adv. Manuf. Polym. Compos. Sci..

[9] Friedrich K., Almajid A.A. (2013). Manufacturing aspects of advanced polymer composites for automotive applications. Appl. Compos. Mater..

[10] Ishikawa T., Amaoka K., Masubuchi Y., Yamamoto T., Yamanaka A., Arai M., Takahashi J. (2018). Overview of automotive structural composites technology developments in Japan. Compos. Sci. Technol..

[11] Skuse B. The Untapped Potential in Formula 1 Composite Manufacture|CompositesWorld. https://www.compositesworld.com/articles/the-untapped-potential-in-formula-1-composite-manufacture.

[12] Wagih A., Tao R., Lubineau G. (2021). Bio-inspired adhesive joint with improved interlaminar fracture toughness. Compos. Part A Appl. Sci. Manuf..

[13] Gardiner G. Thermoplastic Composite Demonstrators—EU Roadmap for Future Airframes|CompositesWorld. https://www.compositesworld.com/articles/thermoplastic-composite-demonstrators-eu-roadmap-for-future-airframes-.

[14] Khan T., Aziz A.R., Irfan M.S., Cantwell W.J., Umer R. (2022). Energy absorption in carbon fiber honeycomb structures manufactured using a liquid thermoplastic resin. J. Compos. Mater..

[15] Minchenkov K., Vedernikov A., Safonov A., Akhatov I. (2021). Thermoplastic Pultrusion: A Review. Polymers.

[16] Vedernikov A., Minchenkov K., Gusev S., Sulimov A., Zhou P., Li C., Xian G., Akhatov I., Safonov A. (2022). Effects of the Pre-Consolidated Materials Manufacturing Method on the Mechanical Properties of Pultruded Thermoplastic Composites. Polymers.

[17] Ridha M., Tan V.B., Tay T.E. (2011). Traction–separation laws for progressive failure of bonded scarf repair of composite panel. Compos. Struct..

[18] Wang C.H., Gunnion A.J. (2008). On the design methodology of scarf repairs to composite laminates. Compos. Sci. Technol..

[19] Wang C.H., Duong C.N. (2016). Design of scarf and doubler-scarf joints. Bond. Jt. Repairs Compos. Airframe Struct..

[20] Jones J.S., Graves S.R. (1984). Repair Techniques for Celion/LARC-160 Graphite/Polyimide Composite Structures. https://apps.dtic.mil/sti/citations/ADA305211.

[21] Olajide S.O., Kandare E., Khatibi A.A. (2016). Fatigue life uncertainty of adhesively bonded composite scarf joints—An airworthiness perspective. J. Adhes..

[22] Darwish F.H., Shivakumar K.N. (2013). Experimental and Analytical Modeling of Scarf Repaired Composite Panels. Mech. Adv. Mater. Struct..

[23] CWang H., Gunnion A.J. (2015). Optimum shapes for minimising bond stress in scarf repairs. Aust. J. Mech. Eng..

[24] Jen Y.M. (2012). Fatigue life evaluation of adhesively bonded scarf joints. Int. J. Fatigue.

[25] Li J., Yan Y., Zhang T., Liang Z. (2015). Experimental study of adhesively bonded CFRP joints subjected to tensile loads. Int. J. Adhes. Adhes..

[26] Ashcroft I.A., Wahab M.A., Crocombe A.D., Hughes D.J., Shaw S.J. (2001). The effect of environment on the fatigue of bonded composite joints. Part 1: Testing and fractography. Compos. Part A Appl. Sci. Manuf..

[27] Slattery P.G., McCarthy C.T., O’Higgins R.M. (2016). Assessment of residual strength of repaired solid laminate composite materials through mechanical testing. Compos. Struct..

[28] Khan T., Ali M.A., Irfan M.S., Khan K.A., Liao K., Umer R. (2022). Resin infusion process monitoring using graphene coated glass fabric sensors and infusible thermoplastic and thermoset matrices. Polym. Compos..

[29] Obande W., Brádaigh C.M., Ray D. (2021). Continuous fibre-reinforced thermoplastic acrylic-matrix composites prepared by liquid resin infusion—A review. Compos. B Eng..

[30] Khan T., Ali M.A., Irfan M.S., Cantwell W.J., Umer R. (2022). Visualization and investigation of healing mechanism in carbon fiber reinforced Elium^®^ composites. J. Thermoplast. Compos. Mater..

[31] CYCOM 937A|Solvay. https://www.solvay.com/en/product/cycom-937a.

[32] Sheikh-Ahmad J.Y., Almaskari F., Hafeez F. (2019). Thermal aspects in machining CFRPs: Effect of cutter type and cutting parameters. Int. J. Adv. Manuf. Technol..

[33] Irfan M.S., Ali M.A., Khan T., Anwer S., Liao K., Umer R. (2023). MXene and graphene coated multifunctional fiber reinforced aerospace composites with sensing and EMI shielding abilities. Compos. Part A Appl. Sci. Manuf..

[34] Standard Test Method for Plastics: Dynamic Mechanical Properties: In Flexure (Three-Point Bending). https://www.astm.org/d5023-15.html.

[35] Standard Test Method for Flexural Properties of Unreinforced and Reinforced Plastics and Electrical Insulating Materials by Four-Point Bending. https://www.astm.org/d6272-17e01.html.

[36] Khan T., Aydın O.A., Acar V., Aydın M.R., Hülagü B., Bayrakçeken H., Seydibeyoğlu M.Ö., Akbulut H. (2020). Experimental investigation of mechanical and modal properties of Al_2_O_3_ nanoparticle reinforced polyurethane core sandwich structures. Mater. Today Commun..

[37] Khashaba U.A., Othman R., Najjar I.M. (2019). Impact and bending analysis of composite scarf adhesive joints modified with MWCNTs at room and hot temperatures. IOP Conf. Ser. Mater. Sci. Eng..

[38] Khashaba U.A., Aljinaidi A.A., Hamed M.A. (2017). Fatigue and reliability analysis of nano-modified scarf adhesive joints in carbon fiber composites. Compos. B Eng..

[39] Atas C., Akgun Y., Dagdelen O., Icten B.M., Sarikanat M. (2011). An experimental investigation on the low velocity impact response of composite plates repaired by VARIM and hand lay-up processes. Compos. Struct..

[40] Khashaba U.A., Aljinaidi A.A., Hamed M.A. (2015). Analysis of adhesively bonded CFRE composite scarf joints modified with MWCNTs. Compos. Part A Appl. Sci. Manuf..

[41] Khashaba U.A., Najjar I.M. (2018). Adhesive layer analysis for scarf bonded joint in CFRE composites modified with MWCNTs under tensile and fatigue loads. Compos. Struct..

[42] Khan T., Ali M.A., Irfan M.S., Cantwell W.J., Rehan U. (2022). Visualizing pseudo-ductility in carbon/glass fiber hybrid composites manufactured using infusible thermoplastic Elium^®^ resin. Polym. Compos..

[43] Naresh K., Shankar K., Rao B.S., Velmurugan R. (2016). Effect of high strain rate on glass/carbon/hybrid fiber reinforced epoxy laminated composites. Compos. B Eng..

[44] Rathore D.K., Prusty R.K., Mohanty S.C., Singh B.P., Ray B.C. (2017). In-situ elevated temperature flexural and creep response of inter-ply glass/carbon hybrid FRP composites. Mech. Mater..

[45] Din I.U., Naresh K., Umer R., Khan K.A., Drzal L.T., Haq M., Cantwell W.J. (2020). Processing and out-of-plane properties of composites with embedded graphene paper for EMI shielding applications. Compos. Part A Appl. Sci. Manuf..

